# Influence of high‐pressure processing on nutritional composition and bioactive compounds of *Phaseolus coccineus* L

**DOI:** 10.1111/1750-3841.16361

**Published:** 2022-10-31

**Authors:** Araceli Redondo‐Cuenca, Mercedes Martín Pedrosa, Ma Dolores Tenorio Sanz, Alejandra N. Alvarado López, Alejandra Garcia‐Alonso

**Affiliations:** ^1^ Departamento de Nutrición y Ciencia de los Alimentos. Facultad de Farmacia Universidad Complutense de Madrid Madrid Spain; ^2^ Departamento de Tecnología de Alimentos Instituto Nacional de Investigación y Tecnología Agraria y Alimentaria (INIA‐CSIC) Madrid Spain; ^3^ Laboratorio de Toxicología Ambiental, Facultad de Química Universidad Autónoma del Estado de México, Paseo Colón intersección Paseo Tollocan s/n. Col. Residencial Colón Toluca Estado de México México

**Keywords:** bioactive compounds, high‐pressure processing, nutritional composition, *Phaseolus coccineus*

## Abstract

The influence of high‐pressure processing (HPP) prior to cooking on nutritional composition and bioactive compounds content of four varieties of *Phaseolus coccineus* L. was studied. Cooking and HPP+C increased the protein content. However, minerals, total carbohydrates, ciceritol and α‐galactosides were reduced. Fat was not affected by cooking but decreased after HPP+C. For dietary fiber, the behavior observed was different depending on the sample and the treatment applied. HPP+C could be considered a good processing technology to retain the advantageous lower myo‐inositol phosphates isoforms and supply prebiotic oligosaccharides. The trypsin inhibitors activity was lower in the cooked and HPP+C samples; however, there were no significant differences between both thermal treatments. Thus, HPP+C reduced cooking time and preserving or improving the nutritional composition of the beans and their bioactive compounds content.

## 1 INTRODUCTION

The scarlet runner bean (*Phaseolus coccineus* L.) is probably the third‐most important *Phaseolus* species worldwide, after *P. vulgaris* (common bean) and *P. lunatus* (broad bean). *Phaseolus coccineus* L. is a perennial climbing vegetable grown as an annual crop for dry seeds or production of immature pods, and as an ornamental vid (Singh et al., 2016). This species is native to central Mexico, commonly called Ayocote bean, characterized by striking inflorescences in red, white, pink, and bicolor flowers. The seeds are big, 20–25 mm long, 13–14 mm wide, and 8 mm thick, they have different colors: white, black, beige, and purple, and the 1000‐grain weight is between 1000 and 1400 g (Vargas Vázquez et al., 2018). The *P. coccineus* L. was introduced in Spain (Europe) from America during the 18th century, where it is cultivated in La Granja de San Ildefonso (Segovia) and named the Judión de la Granja with appreciated culinary qualities, especially the tenderness of its seed coat and its buttery texture. The bean from La Granja de San Ildefonso was originally food for horses and was not used for human consumption until the 20th century. It became part of Segovian cuisine in 1955 thanks to the chef Tomás Urrialde Garzón (Urrialde et al., 2019). The seeds of the Judion de la Granja are much larger than those of the Ayocotes, the average weight per seed is 3 g, and can be white, purple, or black, the white ones being the most used for human consumption.

It is known that pulses are not usually consumed raw, so various heat‐processing methods are applied prior to consumption to achieve desirable taste, color, and texture and to improve their nutritional properties. Thus, the type of processing is an important factor affecting their composition and characteristics when they are consumed, and it can also be of great importance at an industrial level (Redondo‐Cuenca et al., 2017). Besides, prior t*o* cooking, pulses are usually soaked in water for a few hours to overnight. During soaking, the water is dispersed into the starch granules and protein fractions of beans, which facilitate processes, such as starch gelatinization and protein denaturation during cooking, which soften the texture and reduce cooking time (Siddiq & Uebersax, 2012).

Consumers desire beans with low cooking times to save time and energy. The time it takes to reach the desired endpoint of texture and tenderness is an important quality indicator for breeders, seed distributors, food industries, and consumers. Shorter heating time would improve economies at an industrial scale and increase the acceptability at household scale (Bassett et al., 2021).

High‐pressure processing (HPP) is a nonthermal treatment that has the advantage to minimize undesirable changes produced by traditional thermal treatments, which affect the nutritional and sensory quality of foods, and help keep longer the organoleptic attributes of fresh food (Ferrari et al., 2010). HPP treatment is characterized by three processing parameters: temperature (*T*), pressure (*P*), and exposure time (*t*), which allow great flexibility in the design of the process (Heinz & Buckow, 2010).

The use of HPP before traditional food processing is an innovative technology to recover health‐related compounds, improve health attributes of foods by increasing the bioavailability of micronutrients and phytochemicals, reduce allergenic potential, preserve healthy lipids, and reduce salt intake by increasing salt perception (Barba et al., 2017). In addition, the application of HPP to legumes can reduce processing time and energy and the risk of over‐processing of some parts of voluminous products (Rastogi et al., 2007).

Considering the importance of legumes in human nutrition, this study aimed to evaluate the impact of HPP on cooking time, nutritional composition, and bioactive compounds content of four *P coccineous* L. varieties, three from Mexico and one from Spain, to promote beans consumption. In addition, a comparison of the composition of the selected varieties according to their origin and color was carried out to evaluate their different nutrients content, bioactive compounds content, and processing behavior due to these factors.

## 2 MATERIALS AND METHODS

### 2.1 Materials and chemicals

Four varieties of *P. coccineus* L. were used in this study: three Ayocote beans (white WA, purple PA, and black BA) from Puebla (Mexico) and one white bean from La Granja de San Ildefonso (Segovia, Spain), Judión de La Granja (JG). The samples were stored in closed containers, in a dry environment, and in the dark. All chemicals used were of analytical grade.

### 2.2 Experimental design and samples processing

**FIGURE 1:**
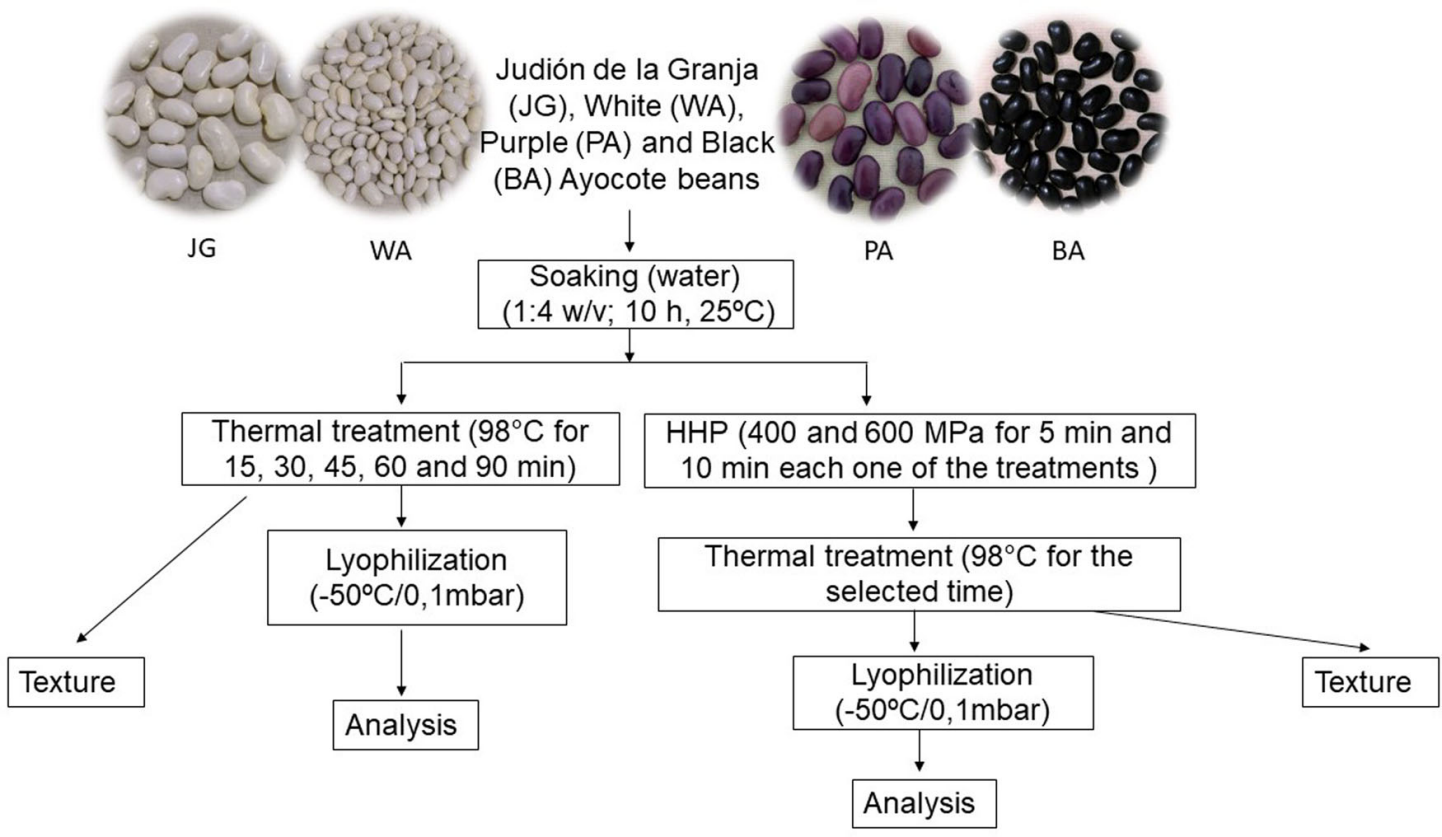
Experimental design

A preliminary study was carried out to select the optimal cooking times through the texture measure of the cooked beans (TA‐XTplus texture analyser, Stable Micro Systems Ltd, Surrey, UK) (Figure 1).

To determine the optimal cooking time, triplicated samples of 250 g of beans corresponding to 250 seeds, except in the case of the JG whose seeds are larger and represent 90 seeds, were used.

Raw beans were soaked in water (1:4 w/v) for 10 h at room temperature. After draining the soaking water using a sieve, one‐fifth of the soaked beans were cooked with 400 mL of water a boiling point (98°C) at atmospheric pressure. During the cooking process, some beans were removed at different times: 15, 30, 45, 60, and 90 min for the hardness evaluation.

The rest of the soaked seeds, divided into four equal parts, were subjected to HPP at the facilities of Hiperbaric (Burgos, Spain). Conditions for the treatments were 400 and 600 MPa for 5 and 10 min, at 25°C. Samples were introduced in polyvinyl packages and vacuum sealed to avoid bubbles during HPP. After HPP treatment, samples were cooked during the most optimal time according to the results obtained for raw samples. In HPP samples, texture was analyzed to determine at which cooking time the same texture was achieved as in the samples without HPP.

Once the optimal cooking and HPP conditions were selected, samples were reprocessed under those conditions. Processed seeds, after the cooking step, were removed from cooking water, freeze‐dried (Telstar model Lyo Quest freeze‐dryer), and milled to pass through a 1 mm sieve. The samples under study were named depending on the treatment as follows: R (raw), C (cooked), and HPP+C (HPP+cooked) for each of the varieties.

### 2.3 Texture hardness

A TA‐XT2i texture analyzer (Stable Micro Systems Ltd, Surrey, UK) was used for the textural analyses of drained cooked beans. The analysis was according to Siqueira et al. (2013). The method employed was the return‐to‐start, measuring force under compression with a 4 mm cylindrical probe, recording the peak of maximum force. Whole beans were axially compressed to 90% of its original height. Force–time curves were recorded at a speed of 1 mm/s and the results corresponded to the average of about 30 measurements of individual cooked grains expressed in Newtons (N).

### 2.4 Nutritional composition

Moisture, ash, protein, and fat contents were determined by AOAC methods (AOAC, 1995). Total carbohydrate content was determined at 630 nm according to the anthrone method (AOAC, 1995), Reducing sugars were quantified at 540 nm by the dinitro‐salicylic acid method as described formerly by Miller (1959). Both colorimetric methods were miniaturized and adapted to a Synergy HTX Absorbance Microplate Reader (BioTek Co., USA). Total dietary fiber (TDF) was determined following the enzymatic‐gravimetric AOAC Method 991.43 (AOAC, 1995)

### 2.5 Mineral composition

Samples were incinerated at a temperature that increased linearly to 550°C for 1 h and then at 500°C for 20 h in a microwave muffle furnace (Milestone MLS‐1200 Pyro, Shelton, CT, USA). The resulting ashes were dissolved in 2 mL of 12 M HCl: 14.5 M HNO_3_ (1:1, v:v) and then diluted to 25 ml with distilled water. An additional dilution was performed for Ca and Mg determination with La_2_O_3_ (expecting a final concentration of 10 g L^−1^) and with CsCl (final concentration of 2 g L^−1^) for Na and K analysis. Mineral element concentrations were measured using a Perkin Elmer Analyst 200 Atomic Absorption Spectrophotometer (MA, USA). This method showed good accuracy (93–99% recoveries), repeatability (below 2%), and reproducibility (less than 4.7%) (Mateos‐Aparicio et al., 2010).

### 2.6 Color

The color was analyzed by a Minolta CR‐200 colorimeter (Osaka, Japan). The parameters analyzed were L* (brightness), a* (redness), and b* (yellowness). The apparatus was calibrated against a standard white plate (*Y* = 86.3, *x* = 0.3165, *y* = 0.3240) and the results obtained were the mean of 10 measurements. The total color difference between the processed samples with respect to the raw samples was evaluated. For this, the following equation was applied:

$$\Delta E={\left[{\left(\Delta L\right)}^{2}+{\left(\Delta a\right)}^{2}+{\left(\Delta b\right)}^{2}\right]}^{1/2}$$

where Δ*L*, Δ*a*, and Δ*b* are the differences of the samples treated with respect to the corresponding raw control sample for those three parameters.

The color difference (Δ*E*) corresponds to a quantity that, from a mathematical formula where the three CIE Lab parameters intervene, allows to approximate the objective color variations between the processed and unprocessed samples to the visual appreciation of them. These differences can be classified as Δ*E* < 0.2 no perceptible difference, 0.2 < Δ*E* < 0. 5 very small difference, 0.2 < Δ*E* < 2 small difference, 2 < Δ*E* < 3 fairly perceptible difference, 3 < Δ*E* < 6 perceptible difference, 6 < Δ*E* < 12 strong difference, and Δ*E* > 12 different colurs, according to the classification of Wang et al. (2014).

### 2.7 Bioactive compounds content

Different bioactive compounds (inositol phosphates, α‐galactosides, and trypsin inhibitors,) were analyzed in the raw and treated Judión and Ayocote samples. Inositol phosphates were analyzed according to Pedrosa et al. (2015) using a HPLC (Beckman System Gold Instrument, Los Angeles, CA, USA) and a PRP‐1 column (150 × 4.1 mm i.d., 5 µm, Hamilton, Reno, NV, USA). Individual inositol phosphates (IP3–IP6) were quantified using an external standard (Sodium phytate; Sigma‐Aldrich, St. Louis, MO, USA). α‐Galactosides were analyzed according to Pedrosa et al. (2015) using a HPLC (Beckman System Gold instrument, Los Angeles, CA, USA) equipped with a refractive index detector and a Spherisorb‐5‐NH2 column (250 × 4.6 mm i.d., Waters, Milford, MA, USA). The individual sugars were quantified by external standards (Sigma‐Aldrich, St. Louis, MO, USA). Trypsin inhibitors were obtained and determined as described by Pedrosa et al. (2015) and trypsin inhibitor units (TIU) were determined using α‐N‐benzoyl‐DL‐arginine‐p‐nitroanilide hydrochloride () as the trypsin substrate. One trypsin unit (TIU) was defined as that which gave a reduction in absorbance units at 410 nm of 0.01 relative to trypsin control reactions, using a 10 mL assay volume.

### 2.8 Statistical analysis

Analyses were performed in triplicate and data are presented as mean ± standard deviation (SD). To establish the statistical significance of differences (*p* < 0.05), ANOVA bifactorial and unifactorial were applied, as well as Duncan's Multiple Range Test. SAS version 9. software was used for this purpose (UCM‐ Servicios Informáticos).

## 3 RESULTS AND DISCUSSION

### 3.1 Selection of bean processing conditions

Hardness of beans processed (Figure 1) by soaking and then cooking for 15, 30, 45, 60, and 90 min was determined as indicated in the Material and Methods section (Table 1). According to Belmiro Tribst and Cristianini (2018) and Siqueira et al. (2013), good texture values for beans range from 2.5 and 4.0 N. In this study, when a sensory comparison of the beans was made for each cooking time, at 60 min a better appearance was observed and a more significant number of whole beans, which were soft but firm to the bite, was seen, and therefore the time was selected as optimal. These conditions occurred when the texture measures ranged from 2.88 to 3.84 N. The same cooking time for *P. vulgaris* L was selected as optimum by Dueñas et al. (2016) using the same cooking conditions.

**TABLE 1:** Texture from HPP and cooked beans

Cooking		Hardness (N)			
Treatment	Time (min)	JG	WA	PA	BA
C	15	13.42 ± 0.79^a^	15.63 ± 3.22^a^	15.79 ± 0.49^a^	16.89 ± 1.30^a^
	30	6.94 ± 0.81^b^	8.51 ± 0.42^b^	6.33 ± 0.18^b^	8.12 ± 0.39^b^
	45	4.11 ± 0.10^c^	4.39 ± 0.28^c^	3.58 ± 0.24^c^	4.10 ± 0.51^c^
	60	2.88 ± 0.13^de^	3.02 ± 0.57^cd^	3.02 ± 0.4^d^	3.84 ± 0.49^c^
	90	1.61 ± 0.23^g^	2.33 ± 0.20^d^	2.40 ± 0.24^efg^	2.37 ± 0.23^e^
HPP+C 1.1	45	2.66 ± 0.24^def^	3.82 ± 0.20^cd^	3.17 ± 0.26^cd^	3.38 ± 0.21^cd^
	60	2.00 ± 0.15^fg^	2.39 ± 0.51^d^	2.23 ± 0.19^fg^	2.48 ± 0.25^e^
HPP+C 1.2	45	2.24 ± 0.04^efg^	3.91 ± 0.15^cd^	2.79 ± 0.21^de^	3.74 ± 0.48^c^
	60	2.04 ± 0.40^fg^	2.33 ± 0.15^d^	2.05 ± 0.11^g^	2.34 ± 0.20^e^
HPP+C 2.1	45	2.68 ± 0.10^def^	3.16 ± 0.27^cd^	2.95 ± 0.28^d^	3.70 ± 0.25^c^
	60	2.36 ± 0.20^ef^	2.60 ± 0.13^cd^	2.02 ± 0.31^g^	2.51 ± 0.34^e^
HPP+C 2.2	45	3.14 ± 0.21^d^	3.07 ± 0.13^cd^	2.70 ± 0.15^def^	3.81 ± 0.20^c^
	60	2.09 ± 0.092^fg^	2.96 ± 0.20^cd^	2.13 ± 0.200^g^	2.60 ± 0.10^de^

*Note*: Mean values of each column followed by different superscript letter significantly differ when subjected to Duncan's multiple range test (*p* < 0.05). Mean ± SD (*n* = 3). HPP+C 1.1 = 400 MPa for 5 min at 25°C, HPP+C 1.2 = 400 MPa for 10 min at 25°C, HPP+C 2.1 = 600 MPa for 5 min at 25°C, HPP+C 2.2 = 600 MPa for 10 min at 25°C.

The application of HPP technology promotes a reduction in the hardness of beans, reducing their cooking time. These changes are related to structural changes in the grain due to the forced entry of water and disruption of cellular structures (Belmiro et al., 2018). In the case of the beans that underwent the HPP+C treatment, a similar range of values (2.68 to 3.70 N) were obtained when the HPP conditions used were 600 MPa for 5 min and 45 min cooking, which means a 25% reduction of the cooking time. Furthermore, the results obtained in both conditions, cooking for 60 min and selected HPP+C for 45 min, showed no statistical differences (*p* < 0.05) but for PA and BA varieties (Table 1).

Therefore, according to the previous results, all parameters in the studied beans were tested after 10 h soaking and then 60 min boiling in cooked samples (C) and HPP 600 MPa for 5 min plus 45 min boiling in HPP+C samples.

### 3.2 Impact of processing on nutritional composition, mineral content, color, and bioactive compounds content

#### 3.2.1 Nutritional composition

The nutritional composition expressed in dry matter of raw, cooked (C), and HPP+C beans studied is presented in Table 2.

**TABLE 2:** Nutritional composition of raw and cooked beans (g/100 g dry matter)

Sample	Protein	Fat	Ash	Total carbohydrates*	Reducing sugars*	Non‐reducing sugars*	Total dietary fiber
JG_R_	22.66 ± 0.41^aC^	2.39 ± 0.10^aB^	4.93 ± 0.09^aA^	52.83 ± 1.32^bA^	2.50 ± 0.11^aB^	50.33 ± 1.21^bA^	24.54 ± 0.78^bA^
WA_R_	17.99 ± 0.15^bC^	2.17 ± 0.07^bcB^	4.40 ± 0.11^bcA^	46.95 ± 0.30^cA^	1.80 ± 0.10^bC^	45.15 ± 0.40^cA^	25.72 ± 0.20^aA^
PA_R_	17.46 ± 0.05^cC^	2.12 ± 0.13^cA^	4.65 ± 0.12^bA^	56.96 ± 2.02^aA^	1.71 ± 0.05^bcC^	55.25 ± 1.97^aA^	23.70 ± 0.56^bAB^
BA_R_	16.3 ± 0.20^dC^	2.34 ± 0.13^abA^	4.15 ± 0.20^cA^	52.73 ± 3.12^bA^	1.59 ± 0.15^cC^	51.14 ± 2.97^bA^	23.69 ± 0.60^bB^
JG_C_	26.75 ± 0.05^aA^	3.08 ± 0.03^aA^	3.82 ± 0.14^aB^	33.39 ± 0.90^bB^	2.8 ± 0.17^bB^	30.59 ± 1.03^cB^	23.09 ± 0.68^bB^
WA_C_	21.2 ± 0.14^bA^	2.62 ± 0.06^bA^	3.05 ± 0.07^bC^	36.09 ± 0.60^aB^	2.52 ± 0.18^bB^	33.57 ± 0.80^abB^	22.65 ± 0.55^bB^
PA_C_	20.67 ± 0.22^cB^	2.08 ± 0.06^cA^	3.16 ± 0.02^bC^	36.28 ± 0.50^aB^	2.63 ± 0.34^bB^	33.65 ± 0.57^aB^	22.86 ± 0.31^bB^
BA_C_	20.79 ± 0.05^cB^	2.55 ± 0.10^bA^	3.08 ± 0.02^bB^	35.34 ± 0.50^aB^	3.13 ± 0.03^aB^	32.21 ± 0.50^bB^	24.33 ± 0.10^aB^
JG_HPP + C_	26.27 ± 0.07^aB^	1.38 ± 0.05^aC^	3.82 ± 0.07^aB^	31.70 ± 0.62^cB^	5.64 ± 0.39^cA^	26.06 ± 0.23^bC^	22.04 ± 0.51^cB^
WA_HPP + C_	20.76 ± 0.13^dB^	1.02 ± 0.06^cC^	3.41 ± 0.03^bB^	36.10 ± 0.60^aB^	5.48 ± 0.16^cA^	30.62 ± 0.84^aC^	25.35 ± 0.56^bA^
PA_HPP + C_	22.31 ± 0.16^bA^	1.23 ± 0.15^abB^	3.49 ± 0.09^bB^	34.21 ± 0,60^bB^	6.82 ± 0.31^bA^	27.39 ± 0.80^bC^	24.86 ± 0.65^bA^
BA_HPP + C_	21.41 ± 0.04^cA^	1.06 ± 0.11^bcB^	2.99 ± 0.06^cB^	34.43 ± 1.38^abB^	7.54 ± 0.40^aA^	26.89 ± 0.57^bC^	26.47 ± 0.23^aA^

*Notes*: Mean values of each column, for each treatment, followed by different superscript letter significantly differ when subjected to Duncan's multiple range test (*p* < 0.05). Mean ± SD (*n* = 3). Mean values of each column, for each variety, followed by different capital superscript letter significantly differ when subjected to Duncan's multiple range test (*p* < 0.05). Mean ± SD (*n* = 3).

Nonreducing sugar are calculated by difference. (*) Expressed in glucose.

JG_R_ = Raw Judión de la Granja, WA_R_ = Raw White Ayocote, PA_R_ = Raw Purple Ayocote, BA_R_ = Raw Black Ayocote,

JG_C_ = Cooked Judión de la Granja, WA_C_ = Cooked White Ayocote, PA_C_ = Cooked Purple Ayocote, BA_C_ = Cooked Black Ayocote, JG_HPP + C_ = HPP+Cooked Judión de la Granja, WA_HPP + C_ = HPP+Cooked White Ayocote, PA_HPP + C_ = HPP+Cooked Purple Ayocote, BA_HPP + C_ = HPP+Cooked Black Ayocote.

**FIGURE 2:**
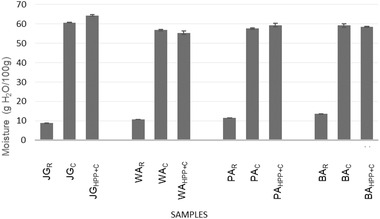
Moisture content of raw and processed beans. Results are expressed as means ± standard deviation (*n* = 3) JG_R_ = Raw Judión de la Granja, WA_R_ = Raw White Ayocote, PA_R_ = Raw Purple Ayocote, BA_R_ = Raw Black Ayocote, JG_C_ = Cooked Judión de la Granja, WA_C_ = Cooked White Ayocote, PA_C_ = Cooked Purple Ayocote, BA_C_ = Cooked Black Ayocote JG_HPP + C_ = HPP+Cooked Judión de la Granja, WA_HPP + C_ = HPP+Cooked White Ayocote, PA_HPP + C_ = HPP+Cooked Purple Ayocote, BA_HPP + C_ = HPP+Cooked Black Ayocote.

The moisture content of the raw samples is much lower than in the processed ones as they are dry seeds (Figure 2). Significant differences can be observed between them with the lowest moisture content in the JG_R_ (8.79 g/100 g) and the highest in the BA_R_ (13.53 g/100 g). Siqueira et al. (2013) studied the effect of cooking on the texture of Carioca beans and reported that the moisture content of the raw samples was between 8.75 and 8.66 g/100 g, more similar to JG_R._

Processing by cooking (C) and HPP+C involves the addition of water, in addition to the water soaked in the soaking. In all cases after soaking, the weight of water absorbed is similar to the weight of the initial beans, that is, the weight doubles. The moisture content of the samples increases after processing, but with a different behavior depending on the type of bean. JG_R_ multiplies its moisture content by seven after the two types of processing, while in the case of Ayocotes, the moisture content after the treatments is between four to five times more, which is striking considering that JG_R_ was the bean with the lowest moisture content (Figure 2). The moisture content for the raw *P. vulgaris* and *P*. *coccineus* studied by Corzo‐Ríos et al. (2020) varied between 8.6 and 11.6 g/100 g, while the moisture content of cooked grains varied between 82.2 and 85.3 g/100 g.

In the raw samples (Table 2), the protein content was very high in all samples, but with statistically significant differences depending on the origin of the sample, so the Spanish one presented a value of 22.66 g/100 g while the Mexican Ayocotes showed a lower content ranging from 16.3 to 17.99 g/100 g and it was observed that white beans had a higher protein content. Malik et al. (2012) indicate that the difference in protein content is associated with the fertilizer application and the growing locations, which influence the yield and protein concentration of some seeds.

The protein values in this study are comparable to the values of 16.7 to 27.2 g/100 g reported by Baptista et al. (2017) in *P. vulgaris* from Mozambique and with the results from Corzo‐Ríos et al. (2020) at 17.7–18.0 g/100 g in Ayocote beans. Mengistu (2017) studied the black *P. coccineus* from Ethiopia and the protein content was 24.63 g/100 g.

The presence of fat (2.39–2.12 g/100 g) was low and more homogeneous between varieties, regardless of their origin, although from a statistical point of view (*p* < 0.05), the JG had a slightly higher fat content than the Mexican samples. Corzo‐Ríos et al. (2020) reported in *P. coccineus*, the purple and brown contents of 2.8 and 3.6 g/100 g, respectively. In the case of Mengistu (2017), the values provided were very low 0.at 90 g/100 g, similar to those reported by Dueñas et al. (2016) in *P. vulgaris* (1.1 g/100 g).

The JG again stood out with the highest mineral content (4.93 g/100 g), the Ayocotes being like each other and lower than the previous one (*p* < 0.05). If these results are compared with those obtained by other authors for the same variety, similar results are observed, 3.9–4.9 g/100 g (Corzo‐Ríos et al., 2020) and 3.99 g/100 g (Mengistu, 2017).

PA showed a high total carbohydrate content of 56.96 g/100 g while JG and BA presented somewhat lower values with no statistically significant differences between the two varieties. In the case of WA, its value was the lowest (46.95 g/100 g). In all samples, the highest carbohydrate content was found to correspond mainly to nonreducing sugars, with the values for reducing sugars being the same or lower than 2.5 g/100 g. The values reported by Dueñas et al. (2016) for *P*. *vulgaris* were higher, at 77.4 mg/100 g. In the study carried out by Mengistu (2017) in *P. coccineus* L the content was higher than that obtained in this work (65.85 g/100 g) but lower than in *P. vulgaris*.

White beans showed the highest fiber content (JG 24.64 and WA 25.72 g/100 g), being significantly different from the colored varieties (PA 23.70 g/100 g and BA 23.69 g/100 g). Values were slightly higher than those reported by Corzo‐Ríos et al. (2020) in purple and brown Ayocote.

When treatments were applied, it was observed that they caused a significant increase in the protein content in all analyzed beans (15–31%) (Table 2). The increases in cooked beans were attributed to solubilization of components and, consequently, as a concentration effect (Wang et al., 2010).

The traditional cooking treatment did not significantly affect the fat content of the colored varieties. However, the HPP+C treatment resulted in a significant decrease in the fat content of all varieties (Table 2). Corzo‐Ríos et al. (2020) point out that in the purple *P. Coccineus*, there was no change in the fat content; however, in the brown beans, there was a significant 20% decrease but too there was a significant increase in the fat content of the white varieties. Wang et al. (2010) reported that cooking resulted in a significant increase (10% approximately) in beans fat content.

With respect to ash, there was a significant decrease in content (Table 2) after both treatments for all the assayed varieties that would result from diffusion of certain minerals into the cooking water. This reduction in ash content has also been observed by Mengistu (2017) and Wang et al. (2010).

Total carbohydrates were reduced by both types of processing (*p* < 0.05) although the decrease was the same regardless of the treatment applied, going from values between 46.95 and 56.96 g/100 g to values between 31.70 and 36.28 g/100 g. As for the reducing and nonreducing fractions of sugars, both treatments increased the reducing sugars and decreased the nonreducing sugars, and in the case of HPP+C, the reducing sugars were significantly increased at the expense of the nonreducing sugars (Table 2).

For TDF, the behaviur observed was very different depending on whether the samples were white or colored and also on the type of treatment applied. Thus, white samples suffered a decrease in their fiber content during cooking but in the HPP+C treatment, their fiber decreased (JG) or was not affected (WA). In the colored samples, the TDF content was not affected by cooking and was increased after the HPP+C treatment.

Corzo‐Ríos et al. (2020) observed changes in the TDF content during cooking and said that it could be due to the formation of resistant starch or to the formation of tannin–protein complexes. Wang et al. (2010) also reported an increase in TDF content from raw beans (14.5−24.5 g/100 g) to cooked beans (20.7−26.7 g/100 g)

#### 3.2.2 Mineral content

In raw samples, results showed that K was the most abundant macro element ranging between 1.30 and 1.63 g/100 g. In contrast, they showed low Na (0.03–0.08 g/100 g). Appreciable concentrations of Mg (0.16–0.20 g/100 g) and Ca (0.09–0.17 g/100 g) were obtained in line with the findings of James et al. (2020) in red beans (0.134 and 0.260 g/100 g, respectively) (Table 3).

**TABLE 3:** Contents of macro‐ and microelements of raw and cooked beans (expressed in dry matter)

Sample	K (g/100 g)	Na (g/100 g)	Ca (g/100 g)	Mg (g/100 g)	Fe (mg/100 g)	Zn (mg/100 g)	Cu (mg/100 g)	Mn (mg/100 g)
JG_R_	1.63 ± 0.06^aA^	0.08 ± 0.00^aA^	0.17 ± 0.01^aA^	0.20 ± 0.01^aA^	6.36 ± 0.37^aA^	3.89 ± 0.29^aA^	0.97 ± 0.30^aA^	1.73 ± 0.05^aA^
WA_R_	1.40 ± 0.02^bA^	0.04 ± 0.01^bA^	0.11 ± 0.00^bC^	0.16 ± 0.01^bA^	5.27 ± 0.20^bA^	2.47 ± 0.18^bAB^	0.69 ± 0.15^aA^	1.28 ± 0.07^bA^
PA_R_	1.44 ± 0.02^bA^	0.03 ± 0.00^bA^	0.09 ± 0.00^cC^	0.18 ± 0.01^bA^	5.36 ± 0.30^bA^	2.64 ± 0.20^bA^	0.67 ± 0.17^aA^	1.25 ± 0.05^bA^
BA_R_	1.30 ± 0.04^cA^	0.03 ± 0.01^bA^	0.11 ± 0.01^bA^	0.19 ± 0.02^abA^	5.25 ± 0.12^bA^	2.62 ± 0.17^bA^	0.61 ± 0.02^aA^	1.07 ± 0.07^cA^
JG_C_	1.23 ± 0.02^aB^	0.03 ± 0.00^abB^	0.17 ± 0.01^aA^	0.16 ± 0.00^aC^	5.32 ± 0.21^aB^	3.33 ± 0.12^aA^	0.44 ± 0.07^aB^	1.79 ± 0.07^aA^
WA_C_	0.98 ± 0.04^bC^	0.02 ± 0.00^bB^	0.13 ± 0.00^bB^	0.13 ± 0.00^cB^	3.73 ± 0.57^bB^	2.16 ± 0.07^cB^	0.17 ± 0.03^bB^	1.24 ± 0.05^bA^
PA_C_	1.03 ± 0.01^bB^	0.03 ± 0.00^aA^	0.11 ± 0.01^cC^	0.14 ± 0.01^bB^	4.75 ± 0.53^aB^	2.49 ± 0.09^bA^	0.22 ± 0.04^bC^	1.21 ± 0.06^bA^
BA_C_	0.99 ± 0.05^bB^	0.03 ± 0.00^abA^	0.13 ± 0.00^bB^	0.14 ± 0.01^bB^	3.51 ± 0,01^bA^	2.24 ± 0,02^bcA^	0.25 ± 0.01^bC^	1.07 ± 0.01^cA^
JG_HPP + C_	1.16 ± 0.01^aB^	0.03 ± 0.00^aB^	0.19 ± 0.01^aA^	0.18 ± 0.01^aC^	4.94 ± 0.30^aB^	3.37 ± 0.30^aA^	0.56 ± 0.10^aAB^	1.55 ± 0.04^aB^
WA_HPP + C_	1.11 ± 0.06^aC^	0.02 ± 0.00^bB^	0.19 ± 0.00^aA^	0.18 ± 0.01^aA^	3.23 ± 0.47^bB^	2.53 ± 0.14^bA^	0.57 ± 0.18^aA^	1.22 ± 0.09^bA^
PA_HPP + C_	1.10 ± 0.06^aB^	0.01 ± 0.00^cB^	0.13 ± 0.01^bA^	0.13 ± 0.01^bC^	3.45 ± 0.25^bB^	2.64 ± 0.19^bA^	0.53 ± 0.04^aA^	1.24 ± 0.09^bA^
BA_HPP + C_	0.94 ± 0.01^bB^	0.02 ± 0.01^bcA^	0.12 ± 0.00^bB^	0.12 ± 0.00^bB^	3.23 ± 0.0^bC^	2.71 ± 0.02^bA^	0.39 ± 0.01^aB^	1.00 ± 0.03^cA^

*Notes*: Mean values of each column, for each treatment, followed by different superscript letter significantly differ when subjected to Duncan's multiple range test (*p* < 0.05). Mean ± SD (*n* = 3). Mean values of each column, for each variety, followed by different capital superscript letter significantly differ when subjected to Duncan's multiple range test (*p* < 0.05). Mean ± SD (*n* = 3).

JG_R_ = Raw Judión de la Granja, WA_R_ = Raw White Ayocote, PA_R_ = Raw Purple Ayocote, BA_R_ = Raw Black Ayocote, JG_C_ = Cooked Judión de la Granja, WA_C_ = Cooked White Ayocote, PA_C_ = Cooked Purple Ayocote, BA_C_ = Cooked Black Ayocote, JG_HPP + C_ = HPP+Cooked Judión de la Granja, WA_HPP + C_ = HPP+Cooked White Ayocote, PA_HPP + C_ = HPP+Cooked Purple Ayocote, BA_HPP + C_ = HPP+Cooked Black Ayocote.

The raw beans are also distinguished by their high content of the microelements Fe and Zn. Fe was the predominant microelement (5.25 to 6.36 mg/100 g). Other authors found similar Fe concentrations in white and kidney beans of 6.2–6.6 mg/100 g (Cabrera et al., 2003). The results of Zn in raw beans were in the range of 2.47–3.89 mg/100 g, within an average value (3.5 mg/100 g) like the one found by Beebe et al. (2000) (2.1–5.4 mg/100 g). In the case of Cu, the values ranged from 0.61 to 0.97 mg/100 g, with no significant differences between the four varieties. For Mn, the values range from 1.07 to 1.73 mg/100 g.

In general, the JG_R_ showed higher content of macro elements than the raw varieties of Ayocote (*p* < 0.05). It should be noted that this trend was the same when microelements were assayed except for Cu, which is the trace element with the lowest levels. It is known that plant variety qualities are mainly affected by genetics (Arriagada et al., 2021) but environmental conditions do influence as well as, for example, the qualities of the same variety may differ greatly when it is grown under different environmental conditions (Rodiño et al., 2007)

Cooking (C) and HPP+C caused a significant decrease in K and Mg contents, of 26% and 22%, respectively, in all beans. In the case of K, the reduction was not affected by the type of treatment. As for Mg, the decreases observed were significant and it was also observed that there were no differences between treatments, except in WA where significant losses were only observed during cooking. Na concentration also suffered a significant decrease in the processed samples compared to the raw ones. The reduced mineral content after processing could be attributed to the minerals leaching into the soaking and cooking water.

Regarding Ca, increases in its concentration were observed only in the Ayocote varieties. In WA_HPP+C_, Ca increased much more than in WA_C_ (*p* < 0.05). In the PA, Ca increased only in the HPP+C treatment (44%) and in the BA the two treatments resulted in a Ca increase of 18% and 9%, respectively. Similarly, increases in Ca had been observed in apple and carob seeds, attributed to the changes induced in the food matrix such as disruption of plant cell walls (Briones‐Labarca et al., 2011). In the other hand, phytic acid is reduced after heat process, which could promote increased mineral extractability and bioavailability (Suarez‐Martínez et al., 2016)

For microelements after the thermal and HPP treatments, it can be observed that in the case of Zn, there are no significant changes. When studying Fe, there was a decrease in the content after both treatments without statistically significant differences when comparing both treatments. In the case of Cu in the JG, there was a decrease in the content without significant differences between the two treatments. In WA and PA, there was only a significant decrease in the values (*p* < 0.05) after cooking the beans. While in BA, there was a decrease after both treatments, but it was higher after cooking (C) than after HPP+C. Finally, when studying the behavior of Mn, there was only a decrease in the content of this mineral in the JG variety when subjected to HPP+C, with no significant changes in Mn values in the other varieties after both treatments. Controversial data are reported in the literature; Ferreira et al. (2014) showed that concentrations of Cu, Fe, and Zn were unaltered by beans cooking, while Ramírez‐Ojeda et al. (2018) showed that element total content decreased in cooked samples with respect to soaked ones, due to a solubilization of the minerals into the water during cooking treatments.

#### 3.2.3 Color

In the raw beans, the similarities between JG and WA in the parameters L*, a*, and b* are clearly visible, but at the same time, there were large differences compared to the colored samples (Table 4). The values of L* and b* in the colored samples were similar, but very different with respect to a*. The results indicated that significant differences in L*, a*, and b* values appeared between beans after both treatments. L* value was decreased after C and HPP+C indicating that grains darkened after processing, except for PA. An increase in the redness (a*) and yellowness (b*) values was observed in JG and WA beans in both treatments. Regarding the colored beans, the behavior is different between them; it was observed that in PA, there was a decrease of a* and an increase of b*. On the other hand, in BA, an increase of a* and b* was observed. From these values, it can be deduced that the white varieties (JG and WA) showed a similar behavior due to the C and HPP+C treatments with respect to the color parameters *L*, a*, and b*, in contrast to the colored beans, which were more heterogeneous.

**TABLE 4:** Color from raw and cooked beans

Sample	L*	a*	b*	ΔE
JG_R_	88.44 ± 0.20^aA^	−0.14 ± 0.01^cB^	10.95 ± 0.10^aB^	
WB_R_	83.65 ± 0.86^bA^	−0.04 ± 0.07^cB^	12.65 ± 0.96^aB^	
PA_R_	37.20 ± 1.98^cA^	15.59 ± 0.62^aA^	−2.57 ± 0.24^bC^	
BA_R_	32.63 ± 0.30^dA^	2.26 ± 0.22^bB^	−2.39 ± 0.13^bB^	
JG_C_	65.69 ± 0.41^aC^	1.02 ± 0.20^cA^	12.91 ± 0.48^aA^	23.06 ± 0.39^aA^
WA_C_	64.81 ± 0.70^aB^	2.41 ± 0.06^cA^	15.59 ± 0.60^aA^	20.15 ± 0.42^aA^
PA_C_	35.77 ± 2.17^bA^	10.55 ± 0.21^aB^	8.33 ± 1.00^bA^	9.81 ± 0.78^bA^
BA_C_	28.13 ± 0.80^cB^	8.74 ± 0.40^bA^	−0.70 ± 0.64^cA^	7.41 ± 1.20^cA^
JG_HPP + C_	69.49 ± 0.20^aB^	0.92 ± 0.21^dA^	9.38 ± 0.35^bB^	19.11 ± 0.60^aB^
WA_HPP + C_	63.70 ± 0.94^bB^	2.66 ± 0.25^cA^	13.12 ± 0.78^aAB^	20.22 ± 0.42^aA^
PA_HPP + C_	36.56 ± 1.50^cA^	11.11 ± 0.45^aB^	2.96 ± 0.30^cB^	8.29 ± 0.39^bB^
BA_HPP + C_	27.59 ± 1.20^dB^	7.15 ± 0.18^bA^	−2.28 ± 0.14^dAB^	6.25 ± 0.35^bA^

*Notes*: Mean values of each column, for each treatment, followed by different superscript letter significantly differ when subjected to Duncan's multiple range test (*p* < 0.05). Mean ± SD (*n* = 3). Mean values of each column, for each variety, followed by different capital superscript letter significantly differ when subjected to Duncan's multiple range test (*p* < 0.05). Mean ± SD (*n* = 3).

Δ*E* = [(ΔL*)2 + (Δa*)2 + (Δb*)2]1/2.

JG_R_ = Raw Judión de la Granja, WA_R_ = Raw White Ayocote, PA_R_ = Raw Purple Ayocote, BA_R_ = Raw Black Ayocote, JG_C_ = Cooked Judión de la Granja, WA_C_ = Cooked White Ayocote, PA_C_ = Cooked Purple Ayocote, BA_C_ = Cooked Black Ayocote, JG_HPP + C_ = HPP+Cooked Judión de la Granja, WA_HPP + C_ = HPP+Cooked White Ayocote, PA_HPP + C_ = HPP+Cooked Purple Ayocote, BA_HPP + C_ = HPP+Cooked Black Ayocote.

According to the classification of Wang et al. (2014), all the samples subjected to the two types of treatments show a color change that is classified as strong difference (6 < Δ*E* < 12) in the case of colored, and different colors (ΔE > 12) in white samples. This difference may possibly be due to a greater change in the L* parameter in the case of JG and WA.

The beans treated by HPP+C underwent a less color change than the cooked beans, indicating that HPP+C treatment can minimize color changes in some varieties. These results are similar to those presented by Inanoglu et al. (2021) when studying the effect of HPP (600 MPa at 25° C for 10 min) and microwave‐assisted thermal pasteurization (70° C for 2 min) on green beans.

#### 3.2.4 Bioactive compounds

Although there are some papers about the chemical composition of *P. coccineus*, to the best of our knowledge, no studies on the effect of HPP on the phytic acid, α‐galactosides, and trypsin inhibitors content of this bean are available until now.

Table 5 shows the bioactive compounds myo‐inositol phosphates, ciceritol, α‐galactosides, and trypsin inhibitors in the raw, cooked, and HPP+C JG and Ayocote samples.

**TABLE 5:** Inositol phosphates (mg/g d.m), ciceritol (mg/g d.m.), raffinose family oligosaccharides (raffinose, stachyose) (mg/g d.m.), and trypsin inhibitors (TIU/mg d.m) content in different raw and processed *Phaseolus coccineus* samples

Sample	IP3	IP4	IP5	IP6	Total IP	Ciceritol	Raffinose	Stachyose	Total galactosides	Trypsin inhibitors
JC_R_	0.23 ± 0.01^aA^	0.25 ± 0.01^aA^	0.66 ± 0.15^aA^	4.97 ± 0.42^fA^	6.11 ± 0.49^deA^	12.01 ± 0.42^fA^	3.14 ± 0.06^deA^	42.29 ± 0.39^gA^	45.43 ± 0.38^hA^	63.55 ± 1.17^eA^
WA_R_	0.21 ± 0.00^abA^	0.38 ± 0.03^efA^	1.04 ± 0.03^efA^	1.75 ± 0.12^aA^	3.39 ± 0.12^aA^	12.61 ± 0.52^fA^	4.31 ± 0.24^fA^	37.86 ± 1.39^fA^	42.17 ± 1.61^gA^	37.60 ± 0.83^cA^
PA_R_	0.21 ± 0.00^abA^	0.25 ± 0.01^aA^	0.55 ± 0.05^abA^	3.38 ± 0.06^cA^	4.38 ± 0.09^bA^	12.27 ± 1.34^fA^	3.88 ± 0.60^gA^	34.62 ± 2.37^eA^	38.50 ± 2.93^fA^	39.48 ± 0.61^dA^
BA_R_	0.21 ± 0.00^aA^	0.26 ± 0.01^abA^	0.50 ± 0.01^aA^	2.47 ± 0.09^bA^	3.43 ± 0.07^aA^	11.79 ± 0.25^fA^	3.19 ± 0.28^deA^	31.42 ± 0.23^dA^	34.61 ± 0.27^eA^	36.07 ± 1.44^bA^
JG_C_	0.21 ± 0.01^bB^	0.31 ± 0.04^bcB^	1.35 ± 0.03^gB^	7.10 ± 0.09^iB^	8.97 ± 0.10^hB^	8.64 ± 0.32^deB^	3.37 ± 0.34^eA^	40.82 ± 1.62^gA^	44.18 ± 1.45^ghB^	0.26 ± 0.03^aB^
WA_C_	0.20 ± 0.00^aA^	0.43 ± 0.03^aA^	1.40 ± 0.09^gB^	3.62 ± 0.12^cdB^	5.65 ± 0.19^cdB^	7.07 ± 0.38^abB^	2.84 ± 0.09^cdB^	27.64 ± 0.53^cB^	30.48 ± 0.60^dB^	0.20 ± 0.04^aB^
PA_C_	0.21 ± 0.00^abA^	0.30 ± 0.02^bcB^	1.04 ± 0.02^defB^	4.13 ± 0.12^eB^	5.68 ± 0.14^cdeB^	6.99 ± 0.31^aB^	2.61 ± 0.21^bcB^	27.12 ± 0.85^cB^	29.73 ± 1.04^cdB^	0.23 ± 0.01^aB^
BA_C_	n.d.	0.36 ± 0.03^deB^	1.09 ± 0.18^fB^	4.11 ± 0.08^eB^	5.57 ± 0.23^cB^	8.70 ± 0.29^deB^	2.87 ± 0.20^cdB^	30.24 ± 1.12^dA^	33.10 ± 1.21^eB^	0.21 ± 0.05^aB^
JG_HPP+C_	0.21 ± 0.01^abC^	0.36 ± 0.03^deC^	0.90 ± 0.03^cdC^	6.23 ± 0.18^hC^	7.71 ± 0.19^gC^	8.92 ± 0.43^eB^	1.67 ± 0.24^aC^	26.58 ± 0.21^cC^	28.25 ± 0.38^bcC^	0.36 ± 0.02^aB^
WA_HPP+C_	0.21 ± 0.00^aA^	0.49 ± 0.06^cC^	1.44 ± 0.11^gA^	4.04 ± 0.16^eC^	6.18 ± 0.28^eC^	7.47 ± 0.51^abcB^	2.19 ± 0.25^bC^	24.30 ± 0.94^bC^	26.50 ± 1.05^bC^	0.23 ± 0.01^aB^
PA_HPP+C_	0.21 ± 0.00^abA^	0.32 ± 0.02^cdB^	0.95 ± 0.01^cdeB^	5.55 ± 0.20^gC^	7.03 ± 0.21^fC^	7.99 ± 0.29^bcdC^	3.05 ± 0.25^deC^	23.53 ± 0.65^bC^	26.58 ± 0.82^bC^	0.31 ± 0.07^aB^
BA_HPP+C_	0.20 ± 0.00^aA^	0.27 ± 0.03^bcA^	0.79 ± 0.03^cC^	3.53 ± 0.12^deB^	4.79 ± 0.12^cB^	8.14 ± 1.33^cdeB^	3.23 ± 0.41^deA^	20.91 ± 2.33^aC^	24.15 ± 2.71^aC^	0.26 ± 0.01^aB^

*Notes*: Mean values of each column, for each treatment, followed by different superscript letter significantly differ when subjected to Duncan's multiple range test (*p* < 0.05). Mean ± SD (*n* = 3).

Mean values of each column, for each variety, followed by different capital superscript letter significantly differ when subjected to Duncan's multiple range test (*p* < 0.05). Mean ± SD (*n* = 4).

JG_R_ = Raw Judión de la Granja, WA_R_ = Raw White Ayocote, PA_R_ = Raw Purple Ayocote, BA_R_ = Raw Black Ayocote, JG_C_ = Cooked Judión de la Granja, WA_C_ = Cooked White Ayocote,

PA_C_ = Cooked Purple Ayocote, BA_C_ = Cooked Black Ayocote, JG_HPP + C_ = HPP+Cooked Judión de la Granja, WA_HPP + C_ = HPP+Cooked White Ayocote,

PA_HPP + C_ = HPP+Cooked Purple Ayocote, BA_HPP + C_ = HPP+Cooked Black Ayocote.

Regarding the myo‐inositol phosphates (IP) content, different isoforms (IP3–IP6) were present in all the samples. Phytic acid or myo‐inositol hexakisphosphate or IP6 was always the main form in raw and processed samples, followed by IP5 > IP4 > IP3.The highest total IP content in the raw samples corresponded to JG_R_ (6.11 mg/g) and the lowest one to the BA_R_ and WA_R_ (3.39 and 3.43 mg/g, respectively). The IP6 content ranged from 1.75 (WA_R_) to 4.97 mg/g (JG_R_). Corzo‐Ríos et al. (2020) reported higher amounts of phytic acid (10.30 mg/g) in two Ayocote samples (purple and brown); whereas Alcázar‐Valle et al. (2020) reported lower amount (0.47 mg/g) of phytic acid (IP6) in a sample of *P. coccineus*. This huge difference can be attributed to different varieties of Ayocote as well as the methods used for these authors and the present study (Megazyme kit versus HPLC analysis). The cooking process increased significantly (*p* < 0.05) the IP content of all the isoforms compared to content of their corresponding raw samples, except for the IP3 content of almost all the samples and the IP4 content of the WA_R_/WA_C_ pair. The IP6 content of the cooked samples ranged from 7.10 mg/g for JG_C_ to 3.14 mg/g for BA_C_. In the JG_C_, the observed increases were around a 43% for IP6, 104% for IP5, 22% for IP4, and 5% for IP3, being the total IP increase of 47%. WA_C_ showed the highest increase of the total IP content (67%) mainly due to the increase observed for IP6 (106%). PA_C_ and BA_C_ showed a similar increase of around 29% corresponding to the highest increase of the IP5 form (90 and 98%, respectively). These different percentages could be related to the microstructure of each variety. The increases observed after cooking could be attributed to the breaking of insoluble complexes of phytates with cations and proteins that can release the different isoforms making them available for the extraction during the analysis. On the contrary, Corzo‐Ríos et al. (2020) reported a reduction of 10% of phytic acid content after cooking at 120°C for 20 min for two Ayocote samples. Linsberger‐Martin et al. (2013) reported a reduction up to 48% in the IP6 content of soaked (24 h) and cooked (up to 80 min) peas and white bean samples. This difference could be due to the differences in the thermal treatment since the Ayocote samples were soaked 10 h and cooked 60 min. Compared to the raw seeds, the samples processed by HPP+C showed a significant (*p* < 0.05) increase of the IP4–IP5 forms, while the IP3 content was reduced. It is known that inositol hexakisphosphate, phytate, or phytic acid (IP6) form complexes with some minerals (iron, zinc, or calcium), which negatively affects their absorption. However, the lower phosphorylated forms (IP–IP4) are considered to have a significant function in human health, boosting the assimilation of minerals and preventing the formation of kidney stones, and performing key roles in some disorders, such as type 2 diabetes, some types of cancer, and irritable bowel syndrome (Pedrosa et al., 2021). Regarding these results, HPP+C might be considered a good processing technology to retain the advantageous lower phosphorylated forms in the innovative processed food. The highest increase was observed in the WA_HPP+C_ sample, with increases of 82%, 38%, and 29% for the total IP, IP5, and IP4 content. In general, the content of the different IP isoforms in the HPP+C samples was significantly different from the corresponding cooked samples, except for the IP6 content in the pair BA_C_/BA_HPP+C_, for the IP5 and IP4 content in the pair PA_C_/PA_HPP+C_. To the best of our knowledge, there are no studies about the effect of combined HPP and cooking treatments on pulses, except only about HPP; therefore, comparisons cannot be drawn. Belmiro et al. (2020) reported no effect of HPP (50 MPa/1 min or 600 MPa/1 min) on the phytates content of *P. vulgaris* seeds without previous soaking of the seeds. However, Linsberger‐Martin et al. (2013) documented a reduction from 10% to 100% of the phytate content in soybean, peas, and white beans processed by HPP from 100 to 700 MPa/20 min, up to 60°C. These reductions could be related to a higher leaching of IP6 during the previous soaking treatment (24 h). They concluded that it would be necessary for a combination of pressure and time to produce structural changes in the cell wall and reduce significantly the IP6 content.

In all samples, ciceritol (an α‐D‐digalactoside of pinitol that does not belong to the raffinose family of oligosaccharides) and the oligosaccharides of the raffinose family or α‐galactosides, raffinose, and stachyose were determined (Table 5). There were no significant differences in the raw samples for the ciceritol content (on average 12.17 mg/g). Stachyose was the main oligosaccharide of the raffinose family (on average, 36.54 mg/g) while the lowest content corresponded to raffinose (on average, 3.63 mg/g). The JG_R_ and BA_R_ showed the highest and the lowest total α‐galactosides content (45.43 mg/g and 34.61, respectively). Alvarado‐López et al. (2019) reported the sugar and oligosaccharide content of four varieties of Ayocote bean. These authors found lower stachyose content (24.30 mg/g) and a similar amount of raffinose (around 3.37 mg/g) than in the studied samples. They did not find ciceritol in the Ayocote samples, but they reported small amounts of verbascose (0.40 mg/g). Alcázar‐Valle et al. (2020) reported lower amount (3.69 mg/100 g) of oligosaccharides in a sample of *P. coccineus*. As it has been indicated above, this huge difference can be due to different varieties of Ayocote as well as the different methods used. Cooking process produced a significant reduction (*p* < 0.05) of these soluble sugars, ranging from 3.5% for stachyose in JG_C_ to 43.9% for ciceritol in WA_C_. Generally, the highest reductions for the analyzed sugars corresponded to the WA_C_ and PA_C_, after cooking the total α‐galactosides content was reduced from a 3% in JG_R_ to a 28% in WA_C_. These losses are related to the lixiviation of the soluble sugar to the soaking and cooking water as documented by many authors for different legumes; in addition, they reported that not all sugars are affected to the same extent by the treatment, the autoclave process being more effective in reducing oligosaccharides than the traditional cooking process (Pedrosa et al., 2021; Zhang et al., 2019). Linsberger‐Martin et al. (2013) reported a reduction of oligosaccharides by up to 82% in cooked pea and beans (soaked 24 h and cooked up to 80 min). In the case of the study of Lisenberger‐Martin et al (2013), the longer soaking time would allow greater hydration of the seeds; then, the HPP treatment can be more effective in modifying the composition of the seeds. The HPP+C treatment caused a significant (*p* < 0.05) reduction of ciceritol and α‐galactosides content in comparison to both the raw and the cooked samples, these decreases being higher for the raw/HPP+C pairs. It is known that the galactosides are water‐soluble compounds that lixiviate to the cooking water (Pedrosa et al., 2021; Zhang et al., 2019). Taking into account that the cooking water was discarded, the higher reduction in the galactosides content after HPP +C processing (compared to cooking treatment) could be due to the summative effect of the HPP treatment plus the leaching‐out of the galactosides to the cooking water.

On average, the total α‐galactosides content in the HPP+C was 34 and 22% lower than those of the raw and cooked samples, respectively. In comparison to the cooked samples, the JG_HPP+C_ showed the highest reduction for all the analyzed sugars, this reduction of 35% for stachyose and 50% for raffinose. Among the Ayocote samples, the highest reduction of stachyose (31%) was observed in the BA_HPP+C_ sample, while the raffinose content in this sample increased a 13%. As indicated above, no studies on the effect of HPP+C treatment have been found in the literature. In any case, higher reductions were described by Linsberger‐Martin et al. (2013) in HPP split peas and whole white beans (up to 600 MPa, 60 min and 60°C), reaching a decrease of oligosaccharides around 68% in peas and 48% in beans. These differences could be due to the higher soaking time for the samples. Han and Baik (2006) reported that soaking+HPP (1 h, 621 MPa) previously to cooking (30 min) lentils and chickpeas was more effective in the reduction of oligosaccharides than by only soaking. The α‐galactosides, as well as ciceritol, are considered as prebiotic since they are fermented by colonic bacteria with the production of short chain fatty acids (SCFA) and promote the growth of beneficial gut microflora (Pedrosa et al., 2021). Despite there not being a recommended dietary intake of α‐galactosides, Martínez‐Villaluenga et al. (2008) have shown that a dose of 3 g/day of α‐galactosides produces an increase in the intestinal bacteroides, bifidobacterias, and eubacteria without any flatulence discomfort. In this way, one serving (45 g dry weight) of cooked samples can supply from 1.78 g (PA_C_) to 2.65 g (JG_C_) of total α‐galactosides and one serving of HPP+C can supply from 1.45 g (BA_C_) to 1.69 g (JG_C_). Therefore, both the cooked and the HPP+C samples can supply enough α‐galactosides content to exert a prebiotic effect.

The trypsin inhibitors activity in the raw samples ranged from 63.55 TIU/mg in JG_R_ to 36.07 TIU/mg in the BA_R_. The three Ayocote samples showed a similar trypsin inhibitors activity, although the values were significantly different (*p* < 0.05). The purple sample showed the highest activity (39.48 TIU/mg) followed by the white (37.60 TIU/mg) and black (36.07 TIU/mg) raw Ayocotes. These values were higher than that reported by Corzo‐Ríos et al. (2020) for two runner beans (purple and brown) from Mexico (6.90–6.73 TIU/mg) and by Alcázar‐Valle et al. (2020) for a purple Ayocote (0.013 TIU/mg).Trypsin inhibitors are thermolabile compounds, since they are proteins that are total or partially denatured during cooking, losing their activity; thus, the cooking and the HPPC+C treatment reduced their activity by about a 99%, reaching activity values around 0.20–0.36 TIU/mg. In comparison to the raw samples, the cooked and HPP+C samples were significantly different (*p* < 0.05); however, there were no significant differences (*p* > 0.05) between both thermal treatments. Corzo‐Ríos et al. (2020) reported abolishing of the trypsin inhibitor activity after cooking the seeds at 120°C for 20 min. Linsberger‐Martin et al. (2013) eliminated trypsin inhibitor activity in cooked peas and beans. Similarly, red bean powder homogenized with water and treated at 600 MPa, for 5 min at 25°C reduced by 81% the trypsin inhibitor activity (Lee et al., 2018). The optimal conditions of pressure, time, and temperature to reduce trypsin inhibitor activity are variable for each legume (Linsberger‐Martin et al., 2013) probably due to the different inhibitor isoforms with different thermostability present in each legume (Pedrosa et al., 2021).

Even though there is not a recommended amount of protease inhibitor consumption, it is important to note that the traditional Japanese diet contains about 420 protease inhibitor units/day; further, it has been reported that the consumption of the purified protease inhibitor at 25−800 CIU per day during 12 weeks exerted a protective effect against cancer development and doses of up to 2000 CIU/day do not cause health problems in humans (Pedrosa et al., 2021).

## 4 CONCLUSIONS

High‐pressure processing (600 MPa, 5 min, 25°C) prior to cooking is effective in reducing the cooking time up to 25%, which allows maintaining the nutritional value of the studied legumes. HPP+C could be considered a good processing technology to retain the advantageous lower myo‐inositol phosphates isoform. One serving of the HPP+C samples can supply enough α‐galactosides content to exert a prebiotic effect. The trypsin inhibitors activity was lower in the cooked and HPP+C samples; however, there were no significant differences between both thermal treatments. The application of HPP represents an alternative for the food industry, due to reduced cooking time, costs, and energy and preserving or improving the composition of the beans and the presence of potential bioactive compounds.

## AUTHOR CONTRIBUTIONS

**Araceli Redondo‐Cuenca**: Conceptualization; Data curation; Formal analysis; Investigation; Methodology; Project administration; Resources; Supervision; Validation; Visualization; Writing – original draft. **Mercedes Martín Pedrosa**: Formal analysis, Methodology, Writing – original draft. **Ma Dolores Tenorio Sanz**: Formal analysis; Writing – original draft. **López**: Formal analysis; Investigation; Methodology; Writing – original draft. **Alejandra Garcia‐Alonso**: Conceptualization; Investigation; Methodology; Supervision; Validation; Writing – original draft

## CONFLICT OF INTEREST

The authors declare no conflict of interest.
